# Relationship Between Different Risk Factor Patterns and Follow-Up Outcomes in Patients With ST-Segment Elevation Myocardial Infarction

**DOI:** 10.3389/fcvm.2021.633992

**Published:** 2021-05-25

**Authors:** Si Chen, Qianzi Che, Qiwen Zheng, Yan Zhang, Jia Jia, Yiqun Wu, Yong Huo, Dafang Chen

**Affiliations:** ^1^Department of Epidemiology and Biostatistics, School of Public Health, Peking University, Beijing, China; ^2^Department of Basic Research in Clinical Medicine, China Academy of Chinese Medical Sciences, Beijing, China; ^3^Department of Cardiology, Peking University First Hospital, Beijing, China

**Keywords:** latent class analysis, ST-segment elevation myocardial infarction, risk factor, pattern, China population

## Abstract

**Objective:** Few studies have been concerned with the combined influences of the presence of multiple risk factors on follow-up outcomes in AMI patients. Our study aimed to identify risk factor patterns that may be associated with 1-year survival in male patients with ST-segment elevation myocardial infarction (STEMI).

**Methods:** Data were from the China STEMI Care Project Phase 2 (CSCAP-2) collected between 2015 and 2018. A total of 15,675 male STEMI patients were enrolled in this study. Risk factor patterns were characterized using latent class analysis (LCA) according to seven risk factors. Associations between risk factor patterns and follow-up outcomes, including the incidence of major adverse cardiovascular and cerebrovascular events (MACCE) and all-cause death, were investigated by Cox proportional hazard regression analysis.

**Results:** We obtained four risk factor patterns as “young and middle-aged with low levels of multimorbidity,” “middle-aged with overweight,” “middle-aged and elderly with normal weight,” and “elderly with high multimorbidity.” Four patterns had significant differences in event-free survival (*P* < 0.001). As compared with the patients of “young and middle-aged with low levels of multimorbidity” pattern, the risk of incidence of MACCE and all-cause death were increased in patients of “middle-aged with overweight” pattern (All-cause death: *HR* = 1.70, 95% CI:1.29~2.23; MACCE: *HR* = 1.49, 95% CI*:*1.29~1.72), “middle-aged and elderly with normal weight” pattern (All-cause death: *HR* = 3.04, 95% CI: 2.33~3.98; MACCE: *HR* = 1.82, 95% CI: 1.56~2.12), and “elderly with high multimorbidity” pattern (All-cause death: *HR* = 5.78, 95% CI: 4.49~7.42; MACCE: *HR* = 2.67, 95% CI*:* 2.31~3.10).

**Conclusions:** By adopting a Latent Class Analysis Approach, STEMI patients can be characterized into four risk factor patterns with significantly different prognosis. The data is useful for the improvement of community health management in each specific subgroup of patients, which indicates a particular risk factor pattern.

## Introduction

Acute myocardial infarction (AMI) has high morbidity and acute onset and is one of the leading death causes among cardiovascular diseases. In China, the mortality of AMI increased sharply, from 15.46 cases per 100,000 persons in 2002 to 58.69 cases per 100,000 persons in 2016 in urban residents, and 12 cases per 100,000 persons in 2002 to 74.72 cases per 100,000 persons in 2016 in rural residents (1). Till 2030, there'll be 23 million AMI patients in China (2). Numerous studies have shown that risk factors, including aging and obesity smoking, will further increase the mortality of AMI patients (3–5). For example, one study displayed that every additional year of age for AMI patients will lead to an increased risk of 9.3%, with a higher rate of death (6). Other studies revealed that AMI smoker patients increased the risk of mortality by 60% (7). Besides, it is common for AMI patients to have co-morbidities (8). Co-morbidities such as diabetes (9, 10), hypertension (5, 11–13), cerebrovascular disease (14), and chronic renal failure (3, 4) have an independent association with increased mortality in AMI patients. Multi-morbidity (the presence of multiple co-morbidities) is related to poor outcomes. However, prior studies only focused on the association between the incidence of AMI and a single risk factor. Few studies were concentrated on the combined influences of the presence of multiple risk factors and outcomes of AMI patients. Insights into the impact of risk factors patterns on the prognosis of AMI patients may help further define and target the therapeutic strategies to specific groups of patients in an attempt to reduce premature death.

In this study, based on a national healthcare improvement project, we explored the association between risk factor patterns and follow-up outcomes of ST-segment elevation myocardial infarction (STEMI) that is the main type of AMI, with a latent class analysis (LCA) approach. Because that there's a sex difference among risk factors of a STEMI prognosis, we focused on the male participants in our study because of the limited female population included in the cohort.

## Methods

### Study Population

Our study is a prospective study based on a national healthcare improvement project named China STEMI Care Project Phase 2 (CSCAP-2) that was initiated in 2015 and includes three phases. More details about the project have been described previously (15). The analysis cohort was drawn from 20,800 STEMI patients between 2015 and 2018 from 236 hospitals in 23 districts of China. CSCAP-2 was collected during 2015 and 2018. If patients were aged 18 years or over, they were eligible for the study, where multiple admissions for AMI were recorded per person, and only the first admission was included. The admission was within 30 days of onset. We excluded patients for an incomplete baseline questionnaire and physical examination (*n* = 409) or missing key variables, including age, body mass index (BMI), condition of current smoking, diagnosis of hypertension, diabetes, chronic renal disease, and cerebrovascular disease (*n* = 493). All female patients (*n* = 4,277) were excluded. Finally, 15,675 patients were enrolled in the analysis.

### Data Collection Procedures

All enrolled subjects would answer a structured baseline questionnaire that was administered by a trained interviewer. The questionnaire collected various information, including age, sex, smoking habits, diagnosis of hypertension, diabetes, chronic renal disease, and cerebrovascular disease. According to international consensus and distribution tertiles of <45 years, 45–65 years, and ≥65 years, age was categorized as young, middle-aged, and elderly, respectively. The condition of current smoking was identified to be positive given being smoking within 1 month. The diagnosis of diabetes, hypertension, cerebrovascular disease, and chronic renal failure were based on clinical judgmental physical examination such as height and weight were measured based on a standard protocol. BMI was calculated as weight (Kg)/[height(m)]. According to Chinese classifications of normal weight, overweight, and obese, BMI was categorized as <24 kg/m^2^, 24–28 kg/m^2^, and ≥28kg/m^2^. Admission time, Killip classification, and clinical medication of antiplatelet, statins, β-blockers, and anticoagulants were on the basis of inpatient medical records. The Global Registry of Acute Coronary Events (GRACE) score provides an estimate for the probability of death within 6 months of hospital discharge in patients suffering from acute coronary syndrome (ACS). According to the information documented, we calculated the GRACE score in inpatient medical records. The GRACE score was stratified into three categories of low, intermediate, and high as scores of 49–125, 126–154, and 155–319 based on the in-hospital mortality risk for ST-elevation ACS patients.

After discharge from the hospital, patients were followed up from admission to 1 year. Follow-up outcomes included the incidence of major adverse cardiovascular and cerebrovascular events (MACCE) and all-cause death. MACCE includes death, myocardial infarction, stroke, and re-revascularization of percutaneous coronary intervention (PCI) or coronary artery bypass graft (CABG). The outcomes during the hospitalization period were collected at the time of discharge from hospital. After discharge from the hospital, patients were followed up for mortality status in 1 month, 3 months, 6 months, and 1 year through telephone. In the results, the observed maximum follow-up time was 365 days, and the average time was 225 days.

### Statistics Analysis

All data represented a proportion for categorical variables. The chi-square test was performed for the comparison of the difference in categorical variables.

LCA is an unsupervised learning method based on the latent class model (LCM) by maximum likelihood. The basic assumption is that the probability distribution of various responses of explicit variables can be explained by a few mutually exclusive potential category variables, and each category has a specific tendency to choose the response of each explicit variable. It has been generally applied for pattern identification. In our study, LCA latent class definitions were derived to identify the most common patterns of the seven variables for the range from 2 to 10 subgroups. We selected these variables based on their availability in cohorts and their potential prognostic value. The optimal number of subgroups for the risk factor pattern was determined by the first minima of the Bayesian information criterion with a condition to the percentage of patients in each cluster at least 15% of the total. Based on these criteria, the optimal number of clusters was four. The probabilities of membership in each subgroup for every LCA variable were applied to determine the most likely subgroup for each patient. Finally, patients were characterized into four risk-factor patterns by LCA.

The differences in baseline characteristics among risk factor pattern clusters were summarized by conducting chi-squared tests. Then, variable time-to-event comparisons were performed by carrying out a log-rank test. Survival analysis was estimated by the Kaplan–Meier method. Furthermore, Cox proportional hazard regression models were adopted to assess the association between outcomes and risk factor patterns based on LCA subgroups so as to explore the association between risk factor patterns and follow-up outcomes. The result was illustrated by hazard ratio and 95% confidence interval (HR, 95% CI). A two-sided *P*-value of 0.05 was considered statistically significant.

All analyses were performed with R version 3.4.0.

## Results

### Baseline Characteristics of the Study Subject

A total of 15,675 subjects were included in the analysis. The mean age was 59.4(±13.1), and 33.56% were aged 65 and over, where 12.61% of them were obese (BMI ≥ 28 kg/m^2^), and 59.30% of them were currently smoking. The mortality of hypertension, diabetes, chronic renal disease, and cerebrovascular disease were 46.90, 9.72, 18.66, and 1.58%, respectively. Most patients have low Killip classification (78.4% of Level 1). Meanwhile, 93.92% of participates had a high risk of morbidity based on the GRACE score. More details were illustrated in Table 1.

**Table 1:** Characteristics of risk factor patterns.

**Risk factor**		**Global**		**Class 1**		**Class 2**		**Class 3**		**Class 4**		***P***
		***N***	**Per(%)**	***N***	**Per(%)**	***N***	**Per(%)**	***N***	**Per(%)**	***N***	**Per(%)**	
Total		15,675	100	4,891	31.20	2,542	16.22	5,083	32.43	3,159	20.15	
**LCA variables**												
Age	<45	1,821	11.62	146	2.99	3	0.12	1,672	32.89	0	0.00	<0.001
	45–65	8,593	54.82	4,306	88.04	0	0.00	3,411	67.11	876	27.73	
	≥65	5,261	33.56	439	8.98	2,539	99.88	0	0.00	2,283	72.27	
BMI (kg/m^2^)	<24	7,747	49.42	1,744	35.66	1,565	61.57	2,255	44.36	2,183	69.10	<0.001
	24–28	5,951	37.96	2,387	48.80	756	29.74	2,047	40.27	761	24.09	
	≥28	1,977	12.61	760	15.54	221	8.69	781	15.36	215	6.81	
Current smoking	No	6,379	40.70	1,627	33.27	1,797	70.69	1,007	19.81	1,948	61.67	<0.001
	Yes	9,296	59.30	3,264	66.73	745	29.31	4,076	80.19	1,211	38.33	
Hypertension	No	8,323	53.10	413	8.44	162	6.37	4,589	90.28	3,159	100.00	<0.001
	Yes	7,352	46.90	4,478	91.56	2,380	93.63	494	9.72	0	0.00	
Diabetes	No	14,151	90.28	4,246	86.81	1,856	73.01	5,083	100.00	2,966	93.89	<0.001
	Yes	1,524	9.72	645	13.19	686	26.99	0	0.00	193	6.11	
Chronic renal disease	No	12,750	81.34	3,374	68.98	1,937	76.20	4,725	92.96	2,714	85.91	<0.001
	Yes	2,925	18.66	1,517	31.02	605	23.80	358	7.04	445	14.09	
Cerebrovascular disease	No	15,428	98.42	4,789	97.91	2,400	94.41	5,083	100.00	3,156	99.91	<0.001
	Yes	247	1.58	102	2.09	142	5.59	0	0.00	3	0.09	
**Non-LCA variables**												
KILLIP Level	Level I	12,289	78.40	3,945	80.66	1,739	68.41	4,255	83.71	2,350	74.39	<0.001
	Level II	2,094	13.36	607	12.41	463	18.21	545	10.72	479	15.16	
	Level III	539	3.44	140	2.86	153	6.02	93	1.83	153	4.84	
	Level IV	753	4.80	199	4.07	187	7.36	190	3.74	177	5.60	
Admission time	<12 h	3,203	20.43	926	18.93	568	22.34	980	19.28	729	23.08	<0.001
	≥12 h	12,472	79.57	3,965	81.07	1,974	77.66	4,103	80.72	2,430	76.92	
Antiplatelet drugs	Dual	13,465	85.90	4,251	86.91	2,104	82.77	4,469	87.92	2,641	83.60	<0.001
	Others	2,210	14.10	640	13.09	438	17.23	614	12.08	518	16.40	
Stains	No	10,499	66.98	3,299	67.45	1,749	68.80	3,350	65.91	2,101	66.51	0.063
	Yes	5,176	33.02	1,592	32.55	793	31.20	1,733	34.09	1,058	33.49	
β-blockers	No	9,482	60.49	2,774	56.72	1,668	65.62	2,934	57.72	2,106	66.67	<0.001
	Yes	6,193	39.51	2,117	43.28	874	34.38	2,149	42.28	1,053	33.33	
Anticoagulant	No	4,031	25.72	1,194	24.41	837	32.93	1,092	21.48	908	28.74	<0.001
	Yes	3,043	19.41	887	18.14	600	23.60	920	18.10	636	20.13	
Early reperfusion therapy	No	12,632	80.59	4,004	81.86	1,942	76.40	4,163	81.90	2,523	79.87	<0.001
	Thrombolytic therapy only	744	4.75	217	4.44	73	2.87	319	6.28	135	4.27	
	PCI	10,900	69.54	3,480	71.15	1,632	64.20	3,672	72.24	2,116	66.98	
GRACE score	Low	85	0.69	27	0.68	0	0	56	1.32	2	0.09	<0.001
	Intermediate	662	5.39	218	5.53	6	0.35	409	9.61	29	1.23	
	High	11,542	93.92	3,700	93.79	1,733	99.65	3,790	89.07	2,319	98.68	

### Characteristics of Risk Factor Patterns

In terms of the characteristics of their risk factors profile, LCA revealed four distinct groups of patients: class 1, a middle-aged with overweight cluster (mainly consists of middle-aged with overweight patients); class 2, an elderly with high multi-morbidity cluster (consists of a majority of elders with a high prevalence of multi-morbidity patients); class 3, a young and middle-aged with low levels of multi-morbidity cluster (consists of young and middle-aged with low levels of multi-morbidity patients); and class 4, a middle-aged and elderly with normal weight cluster (consists of middle-aged and elderly with a majority of normal weight patients). Multi-morbidity was defined as having two or over two kinds of disease at the same time. Class 2 has the highest possibility for patients experienced with hypertension and cerebrovascular disease. Class 1 has the highest possibility for patients with chronic renal disease and cerebrovascular disease. At the same time, there is a limited possibility for patients in class 3 to have diabetes and cerebrovascular disease. Patients in class 4 have the lowest potential of hypertension (Table 2).

**Table 2:** Conditional probabilities of patients with risk factor patterns and outcomes.

**Risk factors**		**Class 1**	**Class 2**	**Class 3**	**Class 4**
Age	<45	5.49%	1.50%	30.70%	1.79%
	45–65	74.59%	8.10%	69.30%	41.72%
	≥65	19.92%	90.40%	0.00%	56.49%
BMI (kg/m2)	<25	43.15%	61.67%	48.39%	70.61%
	25–28	41.64%	27.54%	36.86%	21.53%
	≥28	15.22%	10.79%	14.75%	7.86%
Current smoking	No	37.19%	71.04%	24.19%	46.47%
	Yes	62.81%	28.96%	75.81%	53.53%
Hypertension	No	25.36%	19.57%	73.36%	83.41%
	Yes	74.64%	80.43%	26.64%	16.59%
Diabetes	No	84.98%	75.02%	100.00%	94.35%
	Yes	15.02%	24.98%	0.00%	5.65%
Chronic renal disease	No	68.82%	75.32%	91.08%	88.52%
	Yes	31.18%	24.68%	8.92%	11.48%
Cerebrovascular disease	No	97.91%	94.57%	99.95%	99.59%
	Yes	2.09%	5.43%	0.05%	0.41%
Latent class probabilities		30.12%	15.33%	30.28%	24.27%

The characteristics according to the classification showed a similar distribution with the probability calculated by the LCM. Patients in class 2 tended to be aged 65 years and over, with 99.88 vs. 8.98%, 0%, and 72.27%, respectively. The majority of their weights were at normal levels (61.57%). Patients in class 1 and class 3 had a higher prevalence of being obese (15.54 and 15.36% vs. 8.69 and 6.81%). Fewer patients in class 2 smoked currently (29.31% vs. 66.73, 80.19%, and 38.33%, respectively). They also had a higher level of multi-morbidity than patients in the other three clusters (Table 1).

### Association With Risk Factor Pattern and Follow-Up Outcomes

**Figure 1:**
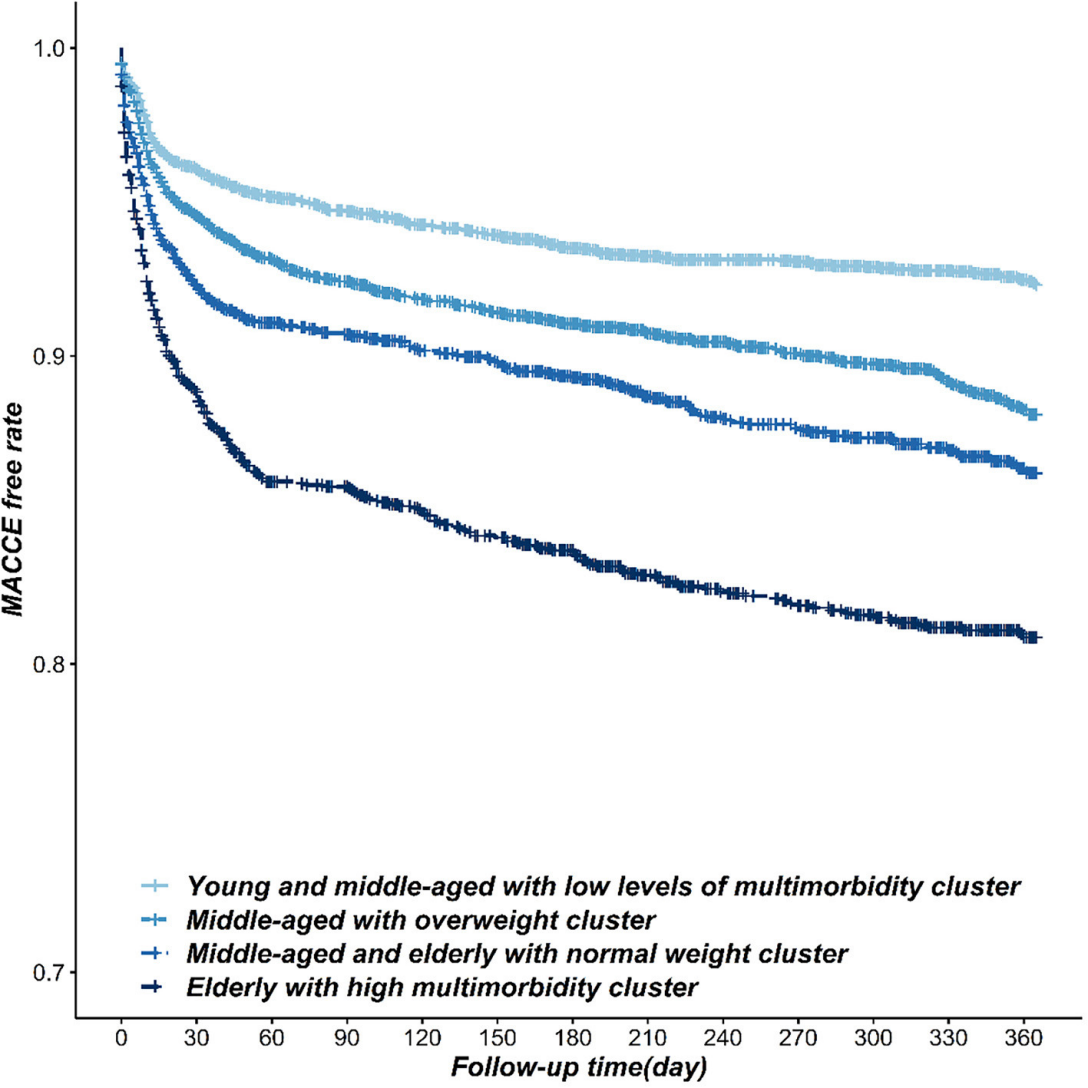
The Kaplan-Meier survival curve of the incidence of MACCE in STEMI patients within different risk factor patterns.

The incidence of MACCE among four-pattern patients had obvious differences (*P* < 0.001), ranked from high to low in order as 15.54% of class 2 patients, 10.76% of class 4 patients, 9.12% of class 1 patients, and 6.06% of class 3 patients. Compared with the patients in the class 3 pattern, the incidence risk of MACCE was significantly higher among class 1 pattern patients (*HR* = 1.49, 95% CI: 1.29~1.72), class 4 pattern patients (*HR* = 1.82, 95% CI:1.56~2.12), and class 2 pattern patients (*HR* = 2.67, 95% CI: 2.31~3.10). After adjusted for education, married status, family history of coronary heart disease, diabetes, hyperlipidemia, admission time, and early reperfusion therapy, the results were similar to the unadjusted model and the adjusted model (Table 3, Figure 1).

**Table 3:** Association with risk factor patterns and outcomes.

**Follow-up outcomes**	**Risk factor pattern**	***N***	**N of events**	**Unadjusted model**				**Adjusted model**			
				**HR**	**95% CI**		***P***	**HR**	**95% CI**		***P***
					**Lower**	**Upper**			**Lower**	**Upper**	
MACCE	Young and middle-aged with low levels of multimorbidity	5,083	308	Ref.				Ref.			
	Middle-aged with overweight	4,891	446	1.49	1.29	1.72	<0.001	1.45	1.21	1.73	<0.001
	Middle-aged and elderly with normal BMI	3,159	340	1.82	1.56	2.12	<0.001	1.45	1.18	1.78	<0.001
	Elderly with high multimorbidity	2,542	395	2.67	2.31	3.1	<0.001	2.01	1.63	2.48	<0.001
All-cause death	Young and middle-aged with low levels of multimorbidity	5,083	82	Ref.				Ref.			
	Middle-aged with overweight	4,891	139	1.7	1.29	2.23	<0.001	1.71	1.18	2.49	<0.001
	Middle-aged and elderly with normal BMI	3,159	154	3.04	2.33	3.98	<0.001	2.13	1.43	3.17	<0.001
	Elderly with high multimorbidity	2,542	233	5.78	4.49	7.42	<0.001	3.77	2.57	5.53	<0.001

*Adjusted Model: adjusted for education, married status, family history of coronary heart disease, hypertension, diabetes, hyperlipidemia, admission time, early reperfusion therapy*.

**Figure 2:**
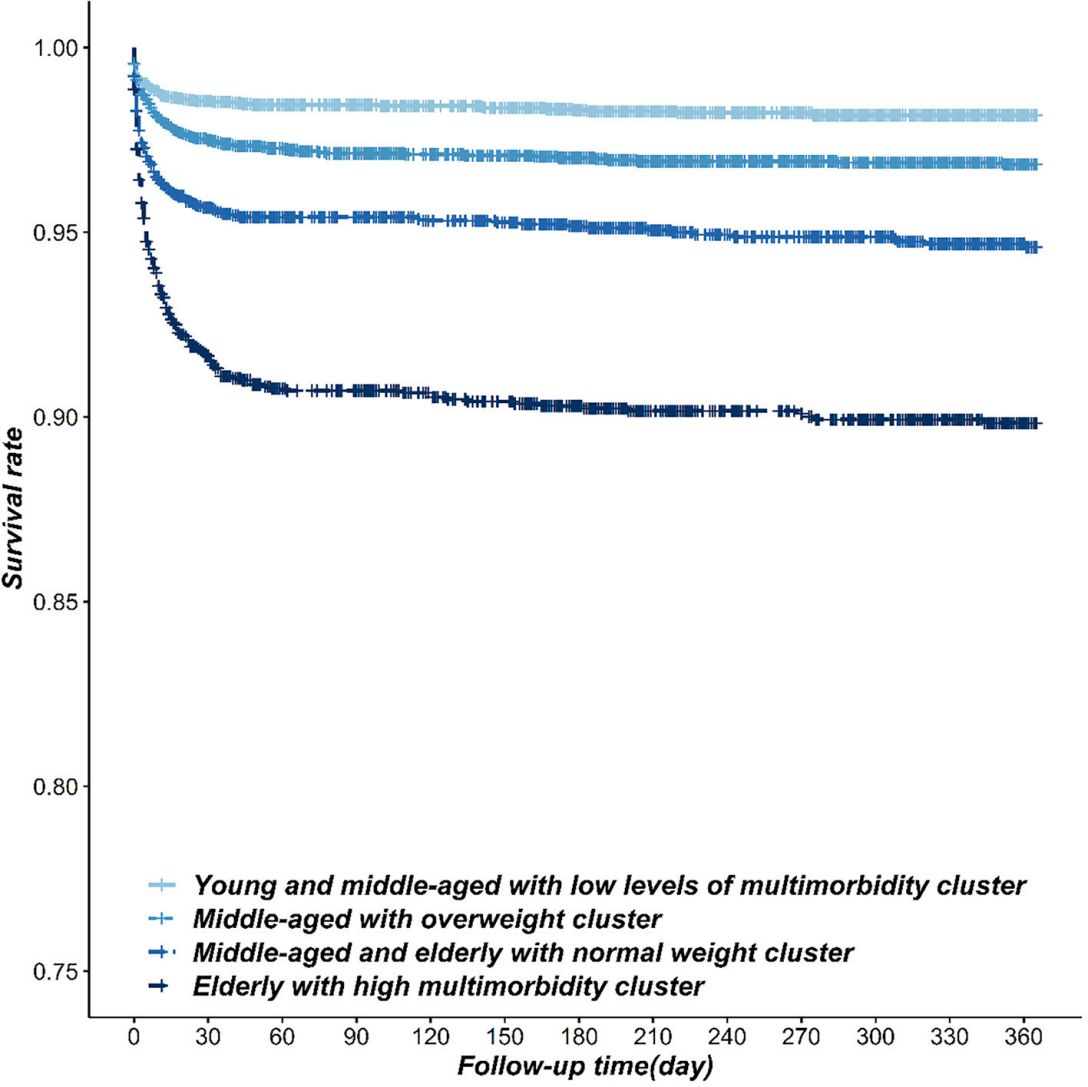
The Kaplan-Meier survival curve of the incidence of all-death cause in STEMI patients within different risk factor patterns.

The incidence of all-cause death among the four-pattern patients had obvious differences (*P* < 0.001), ranked from high to low in order as 9.17% of class 2 patients, 4.87% of class 4 patients, 2.84% of class 1 patients, and 1.61% of class 3 patients. Compared with the patients of the class 3 pattern, the incidence risk of all-cause death was increased in class 1 pattern patients (*HR* = 1.70, 95% CI: 1.29~2.23), class 4 pattern patients (*HR* = 3.04, 95% CI: 2.33~3.98), and class 2 pattern patients (AHR = 5.78, 95% CI: 4.49~7.42). We obtained similar results in the adjusted model after being adjusted for education, married status, family history of coronary heart disease, diabetes, hyperlipidemia, admission time, and early reperfusion therapy (Table 3, Figure 2).

## Discussion

In this study, we discovered that STEMI patients can be divided into four patterns of risk factor from class 1 to class 4 as follows: “young and middle-aged with low levels of multi-morbidity,” “middle-aged with overweight,” “middle-aged and elderly with normal weight,” and “elderly with high multi-morbidity.” The risk of MACCE and death was significantly different between patients with different risk factor patterns from high to low in the order: class 2, class 4, class 1, and class 3 (*P* < 0.05).

We applied LCA to identify the pattern of risk factors. LCA was adopted to identify subgroups to approach the minimum within-subgroup variability and maximize among group variability. It can handle the interrelationship of multiple factors and can be adopted to explore the risk factor pattern for diseases. The external variables are independent in each level of latent classes (16). According to the characteristics of multiple risk factors (external variable characteristics), LCA can identify patients into several potential clusters. Based on 16 co-morbidities, previous studies adopted the LCM to divide 6,480 patients with heart failure in 11 regions of Asia into five patterns of comorbidities. Each pattern's patients have different risks of death (17). Few studies investigated the risk pattern among Chinese STEMI patients.

In our study, we defined age, BMI, hypertension, diabetes, chronic renal disease, and cerebrovascular disease as the risk factors of STEMI mortality. Prior studies proved that aging (5), obesity (5, 13), current smoking (18), hypertension (5, 11–13), cerebrovascular diseases (14), diabetes (19), and chronic renal disease (3, 4) can increase the risk of poor prognosis. However, previous studies tended to concern the influence of a single risk factor and neglected the combined effects of multiple risk factors. Our study concerned the inner relationship of multiple factors by using LCA to classify different risk factor patterns of STEMI patients. Consequently, we identified four risk factor patterns of STEMI patients. Patients in the pattern of class 2 have a higher incidence of hypertension, diabetes, and cerebrovascular disease than those in another three patterns and poorest prognosis. According to previous studies, aging could increase the risk of the incidence of death, MACCE (6), and multi-morbidity (5, 11–13). Furthermore, numerous studies have proved that high multi-morbidity could significantly increase the risk of death and MACCE (20, 21). Similar results have been found in our study. They suggested that more attention should be paid to elderly STEMI patients and multi-morbidity in daily healthcare.

The characteristic of risk factors among the four risk factor patterns illustrated that more current smokers were in the pattern of “young, middle-aged with low levels of multi-morbidity” and “middle-aged with overweight,” accounting for 79.3 and 69.3%, respectively. Both of them had better prognoses than other patterns, indicating a smoker's paradox. Studies have examined that smoking was a protection factor of STEMI patients on prognosis (22). There were two reasons for the paradox. On the one hand, smoking could induce the increase of the activity of CYP1A2, thus increasing the active metabolites of clopidogrel and enhancing the efficacy of antiplatelet drugs (23, 24). On the other hand, patients who smoke were younger than the non-smoker patients. In addition, the obesity paradox also was discovered in our study. The prevalence of obesity was higher among patients in the two patterns with a better prognosis than that in other patterns with poorer prognosis. Some studies presented similar results (25). They tended to attribute the paradox to the uncontrollable confounders (26–28), such as age.

The GRACE score is recommended in international guidelines for risk stratification in ACS. In our study, patients have a low Killip classification with a high risk of death. According to the GRACE score, class 2 and class 4 had a higher risk of death, which met the prediction results using the LCA class in the Cox proportional hazard regression analysis. However, risk score assessment focuses on risk stratification instead of presenting the characteristics of the patient. We could hardly recognize the characteristics of patients with similar risk scores. Understanding the risk factors pattern could exhibit the characteristic of patients directly, thus helping to set personalized interventions in community health management. Otherwise, personalized intervention for each person would cost lots of resources and works where several patterns of intervention suggestions could help. For example, according to the prognosis of each pattern, different follow-up periods were set, and according to the pattern's feature, personalized intervention and health education themes were applied. Patients in class 3 could have longer follow-up periods than patients in class 2 due to different prognoses. Diet management should implement in class 1 patients.

There exist limitations in this study. First, seven risk factors were included in this study for the limitation of data accessibility. Otherwise, as for sex differences in risk factor distributions, we focused on the male patients' risk factor patterns. According to previous researches, the characteristics of STEMI-related risk factors, including smoking habits, BMI, and prevalence of multi-morbidity, had different distributions between males and females (29, 30). Therefore, risk patterns should be investigated under the stratification by sex. However, in our study, the female population is limited. Further analysis of risk factor patterns, including the other risk factors that were related to the prognosis among both of male and female population, should be explored in the future.

## Conclusion

By adopting an LCA approach, STEMI patients can be characterized into four risk factor patterns with significantly different prognoses. The data is useful for the improvement of community health management in each specific subgroup of patients, which indicates a particular risk factor pattern.

## Data Availability Statement

The dataset is not available for public use. Requests to access the datasets should be directed to Dafang Chen, dafangchen@bjmu.edu.cn.

## Ethics Statement

This study was approved by the ethics committee of Peking University First Hospital, and each participant provided written informed consent. We adhered to the principles of the Declaration of Helsinki. The procedures followed were in accordance with institutional guidelines.

## Author Contributions

SC takes responsibility for the statistical analysis and the reliability of our article interpretation. QC helped to complete the study aims and statistical analysis. QZ helped to complete the discussion part in the manuscript. YZ takes responsibility for all aspects of the reliability and freedom from bias of the data presented, who take charge of the data quality control in the China STEMI Care Project Phase 2. JJ helped with data collection. YW helped to revise and perfect the manuscript. YH was the major manager of the China STEMI Care Project Phase 2, who provided the data this manuscript used. DC takes responsibility for all aspects of the reliability and freedom from bias of the data presented and their discussed interpretation, who was the supervisor of the manuscript. All authors contributed to the article and approved the submitted version.

## Conflict of Interest

The authors declare that the research was conducted in the absence of any commercial or financial relationships that could be construed as a potential conflict of interest.
